# Static Magnetic Fields Protect against Cisplatin-Induced Kidney Toxicity

**DOI:** 10.3390/antiox12010073

**Published:** 2022-12-29

**Authors:** Xin Yu, Xinmiao Ji, Yixiang Fan, Biao Yu, Xinyu Wang, Chuanlin Feng, Lei Zhang, Chao Song, Xin Zhang

**Affiliations:** 1 High Magnetic Field Laboratory, Hefei Institutes of Physical Science, Chinese Academy of Sciences, Hefei 230031, China; 2 Science Island Branch of Graduate School, University of Science and Technology of China, Hefei 230036, China; 3 Institutes of Physical Science and Information Technology, Anhui University, Hefei 230601, China; 4 International Magnetobiology Frontier Research Center, Science Island, Hefei 230036, China

**Keywords:** cisplatin, static magnetic field, nephrotoxicity, tumor

## Abstract

Cisplatin is one of the most widely used anti-cancer drugs that can effectively inhibit the growth of multiple types of cancer. However, its clinical application is limited by its severe side effects, especially kidney toxicity, caused by cisplatin-induced oxidative stress, inflammation and kidney cell apoptosis. Here, we found that moderate (a few hundred mT) quasi-uniform static magnetic fields (SMFs) could inhibit cisplatin-induced renal proximal tubular cell death, especially the vertically downward direction SMF. RNA-seq experiments demonstrate that SMFs induced differential gene expressions that are closely associated with oxidative stress, apoptosis, cytokine production, transmembrane transport and DNA repair. In vivo experiments show that SMFs can reduce cisplatin-induced kidney injury in cisplatin-administrated tumor-bearing mice by reducing oxidative stress, inflammation and cell apoptosis. Furthermore, high-dose cisplatin-induced acute nephrotoxicity can be effectively alleviated by SMF treatment of as little as one day, which significantly reduced the reactive oxygen species levels in kidneys and prolonged the mice’s survival. Moreover, the concentration of cisplatin in the kidney was significantly attenuated in SMF-treated mice. Therefore, our study demonstrates the effects of moderate SMFs as a novel physical method to reduce oxidative stress, and revealed their future potential to be used against cisplatin-induced kidney toxicity in cancer treatment.

## 1. Introduction

Since their clinical approval more than 40 years ago, Platinum (Pt) compounds have been widely used as chemotherapy drugs for multiple types of cancer, including cisplatin (cis-diamminedichloroplatinum II; CDDP), which crosslinks purine bases within DNA and interferes with DNA synthesis [1]. At the same time, their clinical administration is limited by severe side effects, especially nephrotoxicity [2,3]. In fact, it has been reported that ~25% patients experience a significant reduction in renal function following cisplatin treatment [4], which has severely affected the quality of life of many patients. However, the most commonly used strategy is currently symptom relief, for example, by hydration and forced diuresis with diuretics or mannitol [5], which unfortunately are often associated with a series of other side effects, including over-diuresis and consequent dehydration [6]. Therefore, it is urgent to look for safer and more effective methods that could prevent and/or alleviate cisplatin-induced side effects and improve the administration of cisplatin.

Cisplatin-induced nephrotoxicity is a complex process that involves various cellular processes, including DNA damage, oxidative stress, inflammation, apoptosis, and mitochondrial dysfunction [7], which have been proposed as potential targets for protective therapeutic interventions [2]. As a noninvasive physical tool, magnetic fields can control the states of unpaired electrons in free radicals, which provide a theoretical physical basis for their regulation of the oxidative stress level [8,9]. In fact, studies have reported that a static magnetic field (SMF) not only can increase the antitumor effects of some types of chemotherapeutic drugs [10], but also can reduce their side effects. For example, our group recently reported that a 9.4 T SMF not only has anti-tumor effects on its own, but also could improve the anti-tumor effect of imatinib mesylate, reduce its toxicity and improve the mice’s mental state [11].

To investigate the effect of SMF on cisplatin-induced kidney injury, we combined cellular assays and animal experiments with two different mouse models to examine two different SMF settings. Our results demonstrate that moderate intensity quasi-uniform SMFs could protect against cisplatin-induced kidney injury by reducing the oxidative stress and cisplatin level in the kidney, which indicates that SMFs have the potential to be used as an adjuvant treatment modality to reduce cisplatin’s side effects in the kidney.

## 2. Materials and Methods

### 2.1. Static Magnetic Field Exposure

We used two types of magnetic fields provided by neodymium N38 permanent magnets. For cellular experiments, we used magnetized or unmagnetized neodymium N38 (length × width × height: 60 mm × 50 mm × 35 mm). The magnets were placed in a cell incubator (Thermo, Waltham, MA, USA) at 37 °C and 5% CO_2_ with either the N or S pole facing the cells. For animal experiments, the magnet plates (length × width × height = 250 mm × 160 mm × 45 mm) were composed of 12 neodymium N38 permanent magnets (length × width × height = 60 mm × 50 mm × 30 mm) purchased from Sans (Nanjing, China). 

To measure the distribution of the magnetic field at cell and mouse locations, a magnet analyzer (FE-2100RD, Forever Elegance, Loudi, China) was used to scan the magnets at 2 mm above the magnets (for cells), or 2.5 cm above the magnetic plates (for mice). Details can be found in our previous study [12]. 

### 2.2. Cell Culture

HK-2 (human kidney-2, a proximal tubular cell line) and breast cancer cell line MDA-MB231 were obtained from the American Type Culture Collection (Manassas, VA, USA). The HK-2 cells were maintained in RPMI-1640 medium (10-040-CV, Corning, CA, USA) containing 10% fetal bovine serum and 1% penicillin/streptomycin. The MDA-MB231 cells were cultured in DMEM medium without L-glutamine (15-017-CVR, Corning, CA, USA), supplemented with 10% fetal bovine serum (FB25015, Clark Bioscience, Richmond, VA, USA), 1% GlutaMAX (35050-061, Gibco, Carlsbad, CA, USA), and 1% penicillin/streptomycin (SV30010, HyClone, Logan, UT, USA). All cells were maintained in a cell incubator (Thermo, Waltham, MA, USA) at 37 °C and 5% CO_2_.

### 2.3. Animal Model

We used two different animal models. Both female BALB/c nude mice and male C57BL/6J mice were purchased from Nanjing GemPharmatech Co., Ltd. (Nanjing, China).

For the chronic model, 30 six-week-old female BALB/c nude mice were subcutaneously injected with MDA-MB231 breast cancer cells. Twenty-four mice with similar tumor sizes were chosen and randomized into four groups (*n* = 6 for each group), one control and three cisplatin-injected groups. For the cisplatin-injected groups, 6 mg/kg of cisplatin (P4394, Sigma, St. Louis, MO, USA) was injected weekly intraperitoneally. The three cisplatin-injected groups were exposed to sham or SMFs for 24 days immediately after cisplatin injection.

For the acute kidney injury mouse model, a total of 60 six-week-old male C57BL/6J mice were divided equally into four groups (*n* = 15 for each group), one control and three cisplatin-injected groups. For the cisplatin-injected groups, 25 mg/kg of cisplatin was injected intraperitoneally to induce acute nephrotoxicity. The three cisplatin-injected groups were exposed to sham or SMFs for five days immediately after cisplatin injection. After sham and SMFs had been exposed for 24 h, five mice per group were randomly selected to test for tissue changes. The survival rate of the remaining mice was recorded for six days. 

### 2.4. Histological Analysis

At the end of the experiments, all mice were dissected to collect organs, which were fixed and processed with formalin to obtain 5-μm-thick sections that were stained with hematoxylin and eosin (HE) and periodic acid–Schiff (PAS). Three to five areas were randomly selected and examined in a blinded way by an independent researcher.

### 2.5. Immunohistochemistry

Kidney section immunohistochemistry was performed using antibodies for F4/80 (70076T, CST, Danvers, MA, USA). All steps were performed according to the manufacturer’s instructions. Three mice per group were randomly examined. Quantitation of staining was evaluated via ImageJ analysis of at least three randomly selected microscopic areas in a blinded way by an independent researcher.

### 2.6. Terminal Deoxynucleotidyl Transferase dUTP Nick-End Labeling (TUNEL) Assay

The TUNEL detection kit (C1098, Beyotime, Shanghai, China) was used to detect apoptotic cells in liver tissue. Cells were stained with 3,3′-diaminobenzidine (DAB) reagent and incubated at room temperature for 5–30 min. We randomly examined kidney tissue from three mice in each group. Liver sections with TUNEL staining were quantitatively analyzed by counting TUNEL positive cells from at least three randomly selected microscopic areas in a blinded way by an independent researcher.

### 2.7. DHE Staining

Reactive oxygen species (ROS) levels in kidney tissue were detected using the Tissue Section Reactive Oxygen Assay Kit (BB-470516, BestBio, Shanghai, China). Washing solution was added to the frozen sections of kidney tissue at room temperature to cover the entire section and left for 5 min before aspirating the washing solution. A dihydroethidium probe was added to the sections, and then the sections were placed in the dark at 37 °C for 60 min to stain. Photographs were taken with a confocal microscope (Olympus, Tokyo, Japan). We randomly tested three mice per group and quantitative analysis was performed via ImageJ analysis of three randomly selected microscopic areas in a blinded way by an independent researcher.

### 2.8. Measurement of Platinum Concentration

After collecting the kidney tissues, 1 mL HNO_3_ (10014518, Sinoreagent, Shanghai, China), 0.75 mL H_2_O_2_ (7722-84-1, Greagent, Shanghai, China), and 0.5 mL HCL (10011018, Sinoreagent, Shanghai, China) were sequentially added and the solution was shaken until the tissues were completely digested. Then, the platinum content in the solution was detected via inductively coupled plasma mass spectrometry (ICP-MS) (PlasmaQuad 3, Waltham, MA, USA).

### 2.9. Cell Viability Analysis

The HK-2 cells were seeded in 96-well plates (1 × 10^4^ cells/mL) and cultured for 24 h. Next, cells were treated with a combination of 20 μM cisplatin and sham, upward or downward SMFs for 24 h before the addition of CCK-8 solution (10 µL/well). After another 30 min of incubation, we examined absorbance at 450 nm with a Microplate Reader (BioRad, Hercules, CA, USA).

### 2.10. Cell Apoptosis Analysis

Cell apoptosis was detected through an Annexin V-FITC Apoptosis Detection Kit (556547, BD Pharmingen™, San Diego, CA, USA). After HK-2 cells (2 × 10^5^ cells/mL) were grown in a 35 mm culture dish for 24 h, cells were treated with a combination of 20 μM cisplatin and sham, upward or downward SMFs for 24 h. Cells were trypsinized and then resuspended in 100 μL 1× binding buffer with 3 μL AnnexinV-FITC and 3 μL PI added. Subsequently, cells were incubated at room temperature in the dark for 40 min; cells were shaken 2–3 times with Vortex during the incubation period. Finally, the samples were supplemented with 400 μL 1× Binding Buffer for detection via flow cytometry.

### 2.11. Cellular ROS Detection

Intracellular ROS levels were detected using the fluorogenic probe 2′, 7′-dichlorofluorescin diacetate (DCFH-DA, D6883, Sigma, St. Louis, MO, USA). HK-2 cells (2 × 10^5^ cells/mL) were seeded in a 35 mm culture dish and cultured for 24 h. Then, 20 μM cisplatin was added to the culture medium while cells were exposed to sham, upward or downward SMFs for 24 h. Cells were harvested and loaded with 10 µM DCFH-DA at 37 °C for 30 min in a medium without serum. The fluorescent intensities were tested using a flow cytometer (CytoFLEX, Beckman Coulter, Brea, CA, USA).

### 2.12. Mice Behavior Tests—Open Field Test (OFT)

The open field test device (SA215, SANS, Nanjing, China) consists of a white plastic box (1000 mm × 1000 mm × 400 mm), divided equally into a total of four closed square spaces, which can accommodate four mice for simultaneous testing. The bottom of each square space was divided into central and peripheral areas. At the beginning of the experiment, the mice were gently placed in the center of the square device and allowed to move freely around the device for 5 min. The entire activity was recorded by a camera mounted on top of the device and connected to a computer, and synchronized data were collected and analyzed using the ANY-Maze Video Tracking System installed on the computer. The experiments and data analysis were performed by two independent researchers in a blinded way.

### 2.13. Western Blotting

Kidney tissues were collected and lysed with Mammalian Protein Extraction Reagent, supplemented with protease inhibitor and phosphatase inhibitor, on ice for 45 min. Subsequently, all lysate was mixed with loading buffer and boiled at 98 °C for 8 min. The proteins were separated using sodium dodecyl sulfate polyacrylamide gel electrophoresis and transferred to a PVDF membrane. Then, the membranes were incubated with primary antibodies organic cation transporter 2 (Oct2) (ab179808, Abcam, Cambridge, UK) and B-actin (HC201-01, Transgene, Beijing, China) overnight at 4 °C, and secondary antibodies for 1 h. ImageJ software was used to quantitatively analyze the expression of proteins.

### 2.14. RNA Extraction Library Construction and Sequencing

HK-2 cells were treated with SMF and cisplatin for 24 h, and then total RNA was extracted using Trizol reagent (15596018, Thermo Fisher, Waltham, MA, USA) following the manufacturer’s procedure. Total RNA quantity and purity were analyzed using Bioanalyzer 2100 and RNA 6000 Nano LabChip Kit (5067-1511, Agilent, Santa Clara, CA, USA). Then, the cleaved RNA fragments were reverse-transcribed to create the cDNA using SuperScript II Reverse Transcriptase (cat.1896649, Invitrogen, Waltham, MA, USA), which was next used to synthesize U-labeled second-stranded DNAs with E. coli DNA polymerase I (cat.m0209, NEB, Ipswich, MA, USA), RNase H (cat.m0297, NEB, Ipswich, MA, USA) and dUTP Solution (cat.R0133, Thermo Fisher, Waltham, MA, USA). An A-base was then added to the blunt ends of each strand, preparing them for ligation to the indexed adapters. After heat-labile UDG enzyme (cat.m0280, NEB, Ipswich, MA, USA) treatment of the U-labeled second-stranded DNAs, the ligated products were amplified with PCR under the following conditions: initial denaturation at 95 °C for 3 min; eight cycles of denaturation at 98 °C for 15 s, annealing at 60 °C for 15 s, and extension at 72 °C for 30 s; and then final extension at 72 °C for 5 min. Last, Illumina Novaseq™ 6000 (LC-Bio Technology CO., Ltd., Hangzhou, China) was used for 2 × 150 bp paired-end sequencing (PE150). Bioinformatic analysis, including Heat map, Gene Ontology (GO) and Venn diagram, was carried out using the OmicStudio tools from https://www.omicstudio.cn/tool (accessed on 20 October 2022). Three samples from each experimental group were used for RNA sequencing.

### 2.15. Statistical Analysis

All statistical analysis was performed using GraphPad Prism version 8. Data from the experiments were shown as means ± SD and were analyzed using Student’s *t*-test. *p* < 0.05 was considered statistically significant.

## 3. Results

### 3.1. SMFs Protect HK-2 Cells from Cisplatin-Induced Cytotoxicity

**Figure 1:**
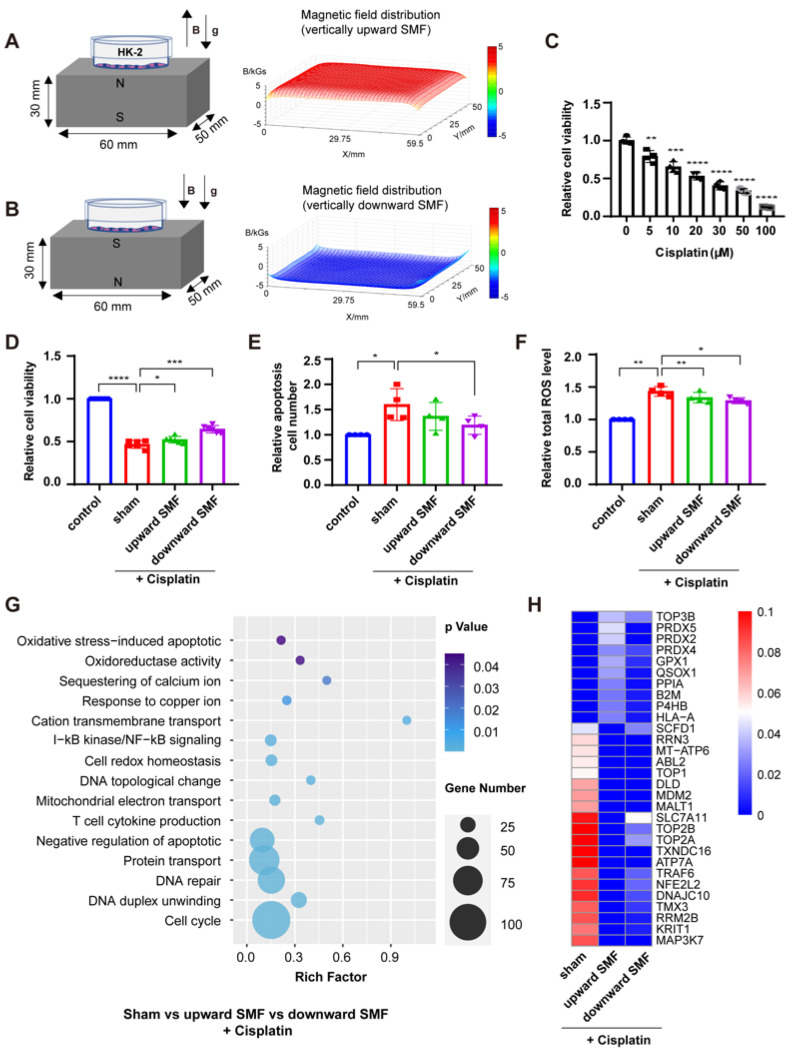
SMFs protect HK-2 cells from cisplatin-induced cytotoxicity. (**A**,**B**) Experimental setup and magnetic field distribution for cells exposed to vertically upward SMF (**A**), or vertically downward SMF (**B**). The SMFs at the cells are ~0.4 T. (**C**) HK-2 cell viability after 24 h cisplatin treatment at different concentrations. Relative cell viability (**D**), apoptosis (**E**), and ROS levels (**F**) of HK-2 cells with or without 20 μM cisplatin and/or SMFs for 24 h. All experiments were repeated more than three times. (**G**) GO term of the differentially expressed genes according to RNA-seq in cisplatin-treated HK-2 cells with or without SMFs. Differential fold change ≥ 2 (i.e., absolute value of log_2_FC ≥ 1) was used as the threshold of change and *p*-value < 0.05 as the criterion for screening differential genes (|log_2_FC| ≥ 1 and *p* < 0.05) in the set comparison group to obtain differentially expressed genes. (**H**) The gene expression heatmap shows the 30 differentially expressed genes in cisplatin-treated cells with or without SMFs. The heatmap was produced using the average transcript expression of three samples in each group. Data are represented as means ± SD. * *p* < 0.05, ** *p* < 0.01, *** *p* < 0.001, **** *p* < 0.0001.

We first used human proximal tubule HK-2 cells to examine the effects of two different SMF directions on cisplatin-induced cytotoxicity in vitro (Figure 1A,B). The magnetic flux density at the cells was about 0.4 T. At this intensity, SMFs alone did not affect HK-2 cell number (Figure S1). As expected, cisplatin decreased HK-2 cell viability in a concentration-dependent manner (Figure 1C), but we found that SMFs could partially rescue the cisplatin-induced reduction in cell viability and apoptosis, as well as the ROS elevation (Figure 1D–F). Since cisplatin-induced cellular ROS elevation is one of the major causes for its cytotoxicity, we hypothesized that SMF may protect against cisplatin damage to HK-2 cells by reducing oxidative stress levels. 

In fact, GO terms from RNA sequencing analysis among cisplatin cells treated with or without SMFs demonstrate that differentially expressed genes are closely associated with oxidative stress processes, while apoptotic processes, cytokine production, transmembrane transport and DNA repair-related genes are also involved (Figure 1G). Moreover, the gene heat map further confirms that multiple oxidative stress process-related genes are involved, including glutathione peroxidase1 (GPX1) and peroxiredoxin family members (PRDX2, PRDX4, PRDX5) (Figure 1H). These results show that these moderate intensity quasi-uniform SMFs can reduce cisplatin-induced oxidative stress in HK-2 cells, which can protect against cisplatin-induced HK-2 cytotoxicity. 

### 3.2. SMFs Improve the Physiological State of Tumor-Bearing Mice Treated with Cisplatin

**Figure 2:**
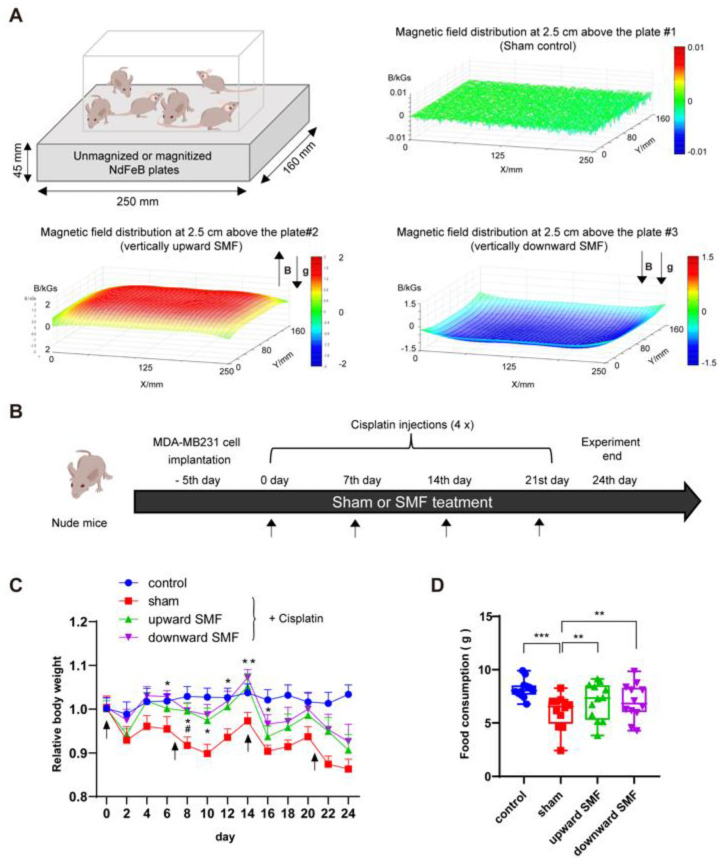
SMFs alleviate cisplatin-induced body weight loss in tumor-bearing mice. (**A**) Magnetic plates providing quasi-uniform SMFs with different directions were used in the animal studies. Schematic illustration of the experimental set up and magnetic flux density distribution at 2.5 cm above the magnetic plates are shown. The SMFs at the mice’s bodies are ~70–220 mT from head to toe. (**B**) Flow chart of the experimental procedure for mice. The relative body weight (**C**) and food consumption (**D**) were measured every two days. There were six mice in each group. Data are represented as means ± SD. * *p* < 0.05, ** *p* < 0.01, *** *p* < 0.001. In (**C**), * *p* < 0.05, ** *p* < 0.01, sham versus downward direction SMF group; # *p* < 0.05, sham versus upward direction SMF group.

Next, we investigated the effect of moderate intensity quasi-uniform SMFs on cisplatin-treated tumor-bearing mice (Figure 2A). MDA-MB231 breast cancer cell-bearing mice were injected with four doses of cisplatin along with sham or SMF exposure 24 h/day for 24 days (Figure 2B). It is obvious that, although cisplatin can significantly reduce tumor size (Figure S2), the mice’s body weight and food consumption were also decreased (Figure 2C,D). However, SMFs significantly alleviated the body weight and food consumption reductions caused by cisplatin (Figure 2C,D), indicating that SMFs improved the physiological state of these cisplatin-treated tumor-bearing mice. 

### 3.3. SMFs Enhanced the Locomotive and Exploratory Activities of Cisplatin-Treated Tumor-Bearing Mice

**Figure 3:**
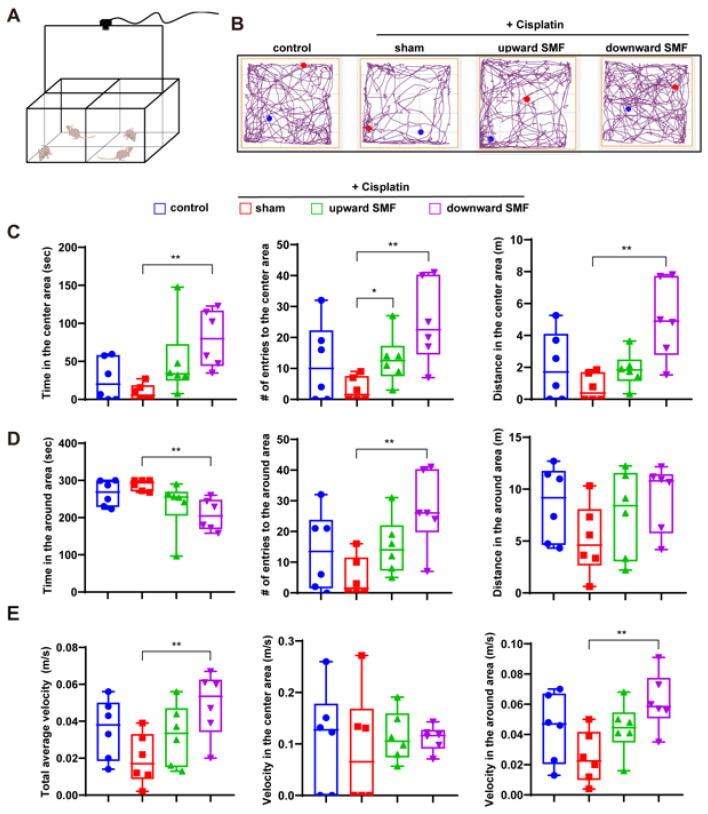
SMFs enhanced the locomotive and exploratory activities of cisplatin-treated tumor-bearing mice. (**A**) Illustration of the OFT experiment setup. (**B**) Representative trajectories of mice in the OFT. The blue and red dots show start and end points, respectively. (**C**) The movement parameters of mice in the center area, including time, number of entries and distance. (**D**) The movement parameters of mice in the surrounding area, including time, number of entries and distance in the surrounding area. (**E**) Velocity of mice during the whole test, in the center or surroundings. There were six mice in each group. Data are represented as means ± SD. Statistical significance is labeled as * *p* < 0.05, ** *p* < 0.01.

In order to fully evaluate the mice’s physiological states, we performed OFT (Figure 3A). The recorded travel path shows that cisplatin-treated mice had much-reduced activity, which is consistent with the side effects of cisplatin. However, the cisplatin-induced activity reduction was significantly alleviated by SMF treatment (Figure 3B). The number of entrances and time spent in the center area are indicators of mice’s exploratory activities because of the natural preference of mice for corners/perimeters. For example, for the control mice, their time spent in the center area was ~26.2 s, but time spent in the surrounding area was ~265.02 s. After cisplatin treatment, their time spent in the center area was ~8.88 s but time spent in the surrounding area was ~288.03 s. It is interesting that SMFs can significantly increase their time and entrances to the center area, indicating enhanced exploratory activities. For velocity, which reflects the locomotive activities of the mice, SMFs also showed a significant improvement effect (Figure 3E). However, it should be noted that the upward SMF was not as effective as the downward SMF (Figure 3C–E). 

### 3.4. SMFs Protect against Cisplatin-Induced Kidney Injury in Tumor-Bearing Mice

**Figure 4:**
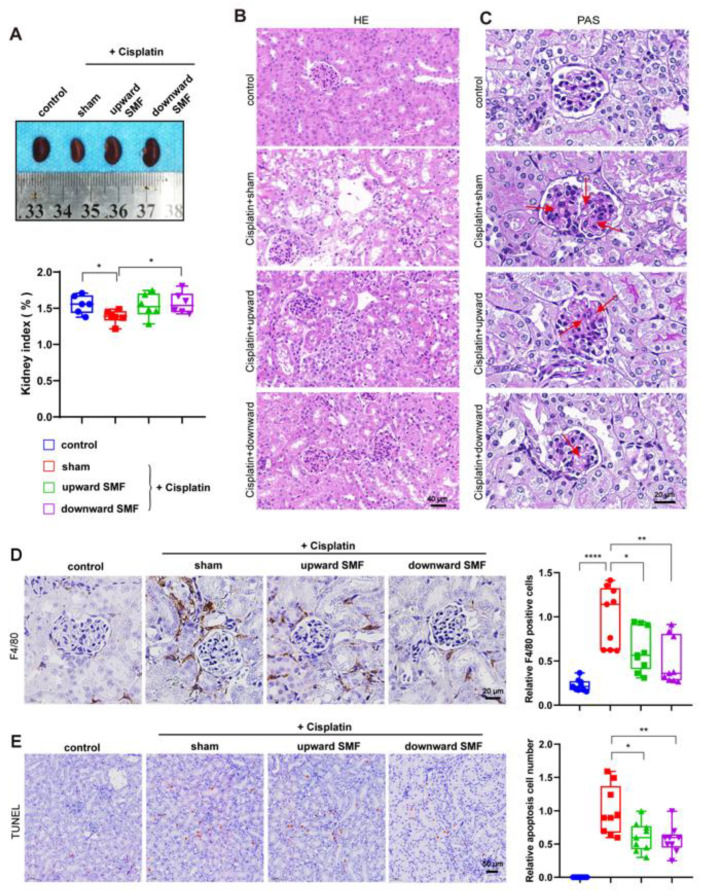
SMFs protect against cisplatin-induced kidney injury. (**A**) Representative images of kidneys and quantification of the kidney index, which is the percentage of kidney weight in the whole-body weight of the mice. (**B**,**C**) Representative HE (**B**) and PAS (**C**) staining of kidney sections. The arrows represent glomerular thylakoid hyperplasia. (**D**) Representative images of F4/80 staining in the mice’s kidneys and quantification of relative F4/80 positive cells. (**E**) Representative images of TUNEL staining in the mice’s kidneys and quantification of relative apoptotic cells. Values show means ± SD. * *p* < 0.05, ** *p* < 0.01, **** *p*< 0.0001.

Next, we examined the mice’s kidneys for the effect of SMFs on cisplatin-induced kidney injury. Our results show that the kidneys of cisplatin-treated mice were 10.98% smaller than those of the control mice (*p* < 0.05), but this adverse effect of cisplatin could be effectively prevented by SMF treatment (Figure 4A). 

We further performed HE staining for the basic structure of the kidney (Figure 4B), and staining to detect glomerular damage (Figure 4C). As shown in HE staining, cisplatin-induced tubular injury, including brush border loss, vacuolar-like degeneration of tubular cells, and partial cell necrosis, can be significantly attenuated by SMFs (Figure 4B). Moreover, cisplatin-induced glomerular thylakoid hyperplasia was also alleviated by SMFs (Figure 4C). 

It is known that the pathophysiology of cisplatin-induced kidney injury involves multiple aspects, including kidney inflammation and vascular injury. Next, we examined renal inflammation via F4/80 staining, which can reflect the infiltration of macrophages into the kidney. It is obvious that cisplatin-treated mice had a significant increase in F4/80 positive cells (*p* < 0.01) (Figure 4D), which can be effectively decreased by both upward and downward SMFs (*p* < 0.05), indicating a decreased level of inflammation. We also performed TUNEL staining to examine apoptotic cells in the mice’s kidneys (Figure 4E). As expected, cisplatin induced a substantial increase in apoptotic cells, but this can be significantly alleviated by SMF treatment (*p* < 0.05). Therefore, these data demonstrate that these moderate intensity quasi-uniform SMFs can reduce cisplatin-induced kidney inflammation and renal cell apoptosis and prevent cisplatin-induced kidney injury in tumor-bearing mice. 

### 3.5. Short-Term SMF Treatment can Effectively Ameliorate Cisplatin-Induced Acute Kidney Injury in Healthy Mice

**Figure 5:**
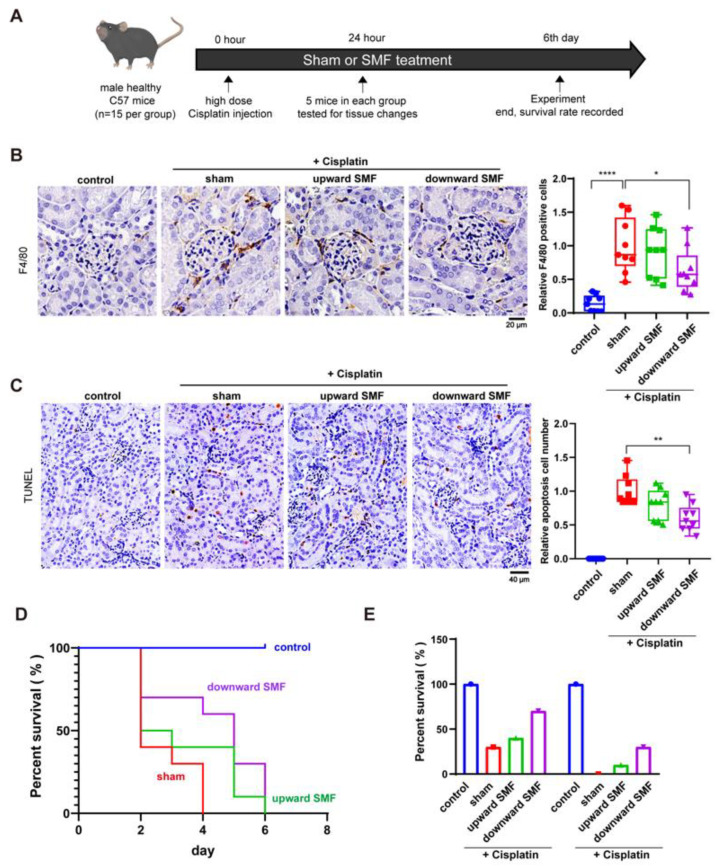
SMFs ameliorate high dose cisplatin-induced acute kidney injury. (**A**) Schematic diagram of high-dose cisplatin-induced acute kidney injury experimental procedures. (**B**) Representative images and quantifications of F4/80 staining. (**C**) Representative images and quantifications of TUNEL staining. (**D**,**E**) Percent survival for mice with or without high doses of cisplatin and/or SMF treatment. Values were expressed as means ± SD, * *p* < 0.05; ** *p* < 0.01, **** *p*< 0.0001.

The above experiments demonstrate that the chronic kidney defects caused by cisplatin can be prevented/reduced by SMF treatment. To investigate whether short term SMF treatment can have an effect on acute conditions, we injected high dose cisplatin at 25 mg/kg into six-week-old male C57BL/6J mice to construct a cisplatin-induced acute kidney injury model (Figure 5A). The mice were simultaneously exposed to sham or SMF conditions (*n* = 15 for each group) for 24 h/day. After 24 h, five mice were taken from each group for tissue examination, while the remaining 10 mice were left in the sham or SMF conditions to record their survival.

It is striking that 24-h short-term SMF treatment can have obvious beneficial effects on cisplatin-induced acute kidney injury. Our HE staining and PAS staining results show that high dose cisplatin induced tubular ectasia, cast formation and tubular degeneration, as well as glomerular atrophy (Figure S3). It is clear that both SMF treatments effectively alleviated these cisplatin-induced kidney injuries, which is consistent with the results from lower dose cisplatin in tumor-bearing mice (Figure 4). In addition, SMFs can also reduce the number of F4/80 positive cells (Figure 5B) and apoptotic cells (Figure 5C), especially the downward SMF. Therefore, our results show that even 24-h short-term SMF treatment can alleviate large-dose cisplatin-induced acute kidney injury.

Besides the five mice that were analyzed for tissue damage, we also used the other 10 mice in each group for survival analysis, which were kept on the sham or SMF condition 24 h/day until death of cisplatin-treated mice. Mice in the control group without cisplatin lived normally until the end of the experiment. It is striking that all mice in the sham group died after four days, while SMF-treated mice had an obviously increased survival rate (Figure 5D,E). 

**Figure 6:**
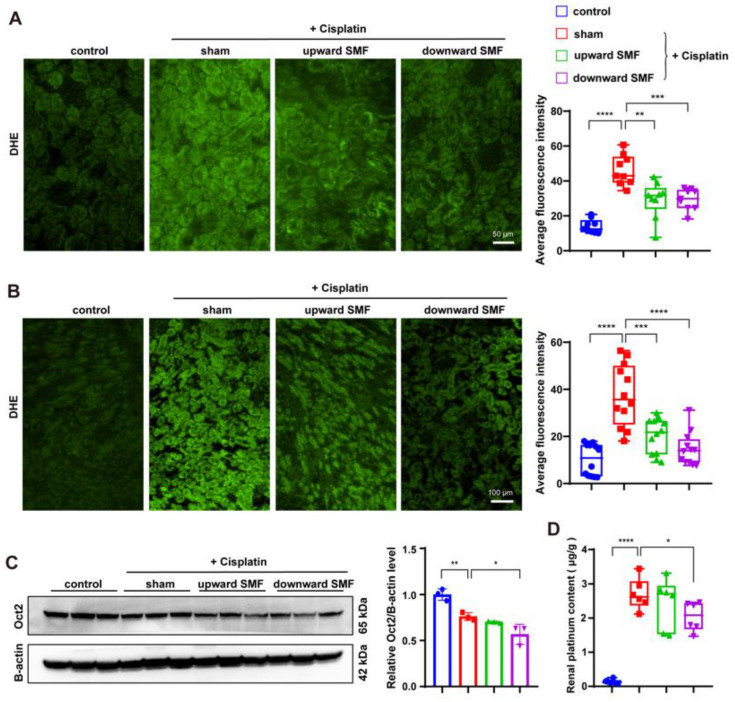
SMFs reduce ROS, Oct2 and cisplatin levels in cisplatin-treated mice’s kidneys. (**A**,**B**) Representative images and quantifications of ROS staining in the kidneys of mice: low-dose multiple-injection cisplatin-treated kidneys (**A**) and high dose single-injection cisplatin-treated kidneys (**B**). (**C**) Representative Western blotting and quantification of Oct2 in low-dose multiple-injection cisplatin-treated kidneys. Three mice were examined for each group. (**D**) ICP-MS was used to measure platinum concentration in low-dose multiple-injection cisplatin-treated kidneys. Values are expressed as means ± SD, * *p* < 0.05; ** *p* < 0.01, *** *p*< 0.001, **** *p*< 0.0001.

To further analyze the reason for the protection effect of SMF on cisplatin-treated mice, we measured the ROS level by using a superoxide anion probe for both batches of mice. For the chronic kidney injury model induced by low-dose cisplatin, the ROS level was 3.27-fold higher than that of the control (*p* < 0.001). However, the ROS level was significantly decreased after SMF treatment (Figure 6A). For acute kidney injury induced by higher-single-dose cisplatin, the cisplatin-induced ROS elevation could also be reduced through SMF treatment (Figure 6B), which confirms that oxidative stress in the kidney was reduced by SMFs. By analyzing the expression of Oct2, the transporter proteins that mediate the transport of cisplatin into the kidney [13], we found that although SMFs did not affect Oct2 levels in HK-2 cells (Figure S4), they could decrease Oct2 levels in cisplatin-treated mice’s kidneys (Figure 6C). Consistently, the accumulated platinum levels in the kidneys were much reduced after SMF treatment, as revealed by ICP-MS (Figure 6D). These results suggest that the preventive effect of SMFs in cisplatin-induced kidney injury may be related to a reduction in cisplatin intake and ROS levels in the kidney. 

## 4. Discussion

**Figure 7:**
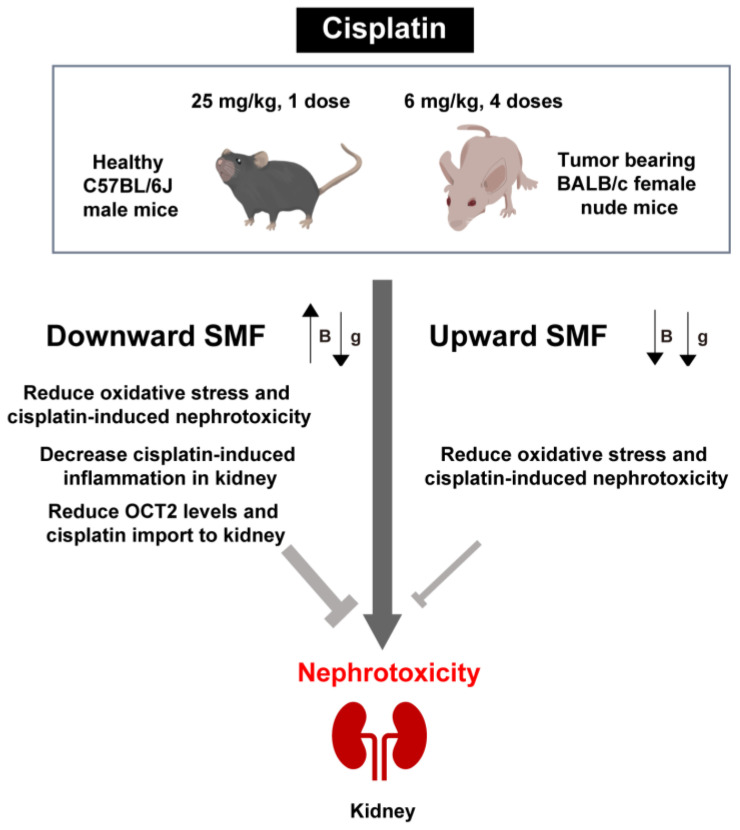
The mechanism of SMF protection against cisplatin-induced renal injury. B, magnetic field direction. g, gravity direction.

Nephrotoxicity is the most significant and dose-limiting side effect encountered in cisplatin-based chemotherapy, imposing a considerable health and economic burden on patients. In the present study, using two different mouse models and two different magnetic settings, we found that moderate intensity quasi-uniform SMFs significantly suppresses cisplatin-induced renal injury in mice by reducing renal ROS level, inflammation, apoptosis, and platinum accumulation, especially for a vertically downward SMF (Figure 7).

It is well known that cisplatin often leads to oxidative stress and subsequent renal pathology [3,14,15]. At the same time, magnetic fields can affect intracellular ROS levels in a field parameter- and cell type-dependent way [16]. Although there is still no conclusive correlation between the exact electromagnetic field parameters and the final effects on ROS levels in living organisms, multiple studies have demonstrated that weak to moderate electromagnetic fields can often reduce ROS levels to modulate physiological and pathological conditions. For example, a SMF alone or in combination with a static electric field can improve type 2 diabetes by decreasing oxidative stress levels in mice [12,17]. Van Huizen et al. found that weak SMFs altered stem cell proliferation and differentiation through decreasing ROS levels [18]. Here, we show that both types of SMF can protect against cisplatin-induced kidney injury by reducing cellular ROS levels.

The exact mechanism by which SMFs regulate ROS levels in living organisms is still not clear. Feng et al. found that exposure to an SMF increased superoxide dismutase (SOD) and decreased nuclear factor erythroid 2-related factor 2 (NRF2) in cells and mice, which may lower oxidative stress [19]. Two studies in plants (one in soybean seeds and the other in broad beans) revealed that SMFs probably affected ROS levels through antioxidant enzymes [20,21]. In addition, the radical pair recombination hypothesis suggests that magnetic fields can affect levels of free radicals by regulating the singlet and triplet states of unpaired electrons [18,22]. However, it is still unclear how SMFs with different parameters generate totally different and unpredictable results on ROS levels. 

Although both upward and downward direction SMFs can reduce the level of oxidative stress in the kidney, the vertically downward direction SMF has a stronger protective effect. Our data show that abnormal pathology of the kidney tissue, as well as inflammation and apoptosis levels, were more significantly reduced by the vertically downward direction SMF. Furthermore, we found that there is lower cisplatin accumulation in the kidneys of mice exposed to the downward SMF due to a decreased Oct2 level, which is involved in the uptake of cisplatin in the kidney [23,24]. In addition, magnetic fields with moderate magnetic induction (10^−3^ T ≤ B < 1 T) have been shown to influence biological systems by interacting with lipids [25], proteins [26,27,28], and glycans [26,29,30,31] of the plasma membrane in an exposure time- and cell type-dependent manner, and the plasma membrane itself is one of the targets of magnetic fields due to its anisotropic and diamagnetic properties [32,33]. However, the exact physical mechanism for the effects of SMFs on Oct2 is still not clear. Moreover, although different-direction SMFs have been shown to have differential bioeffects [34,35,36], the mechanisms for the differential effects of upward and downward SMFs on inflammation and Oct2 are still not clear, which is the major limitation of our study. 

It should also be mentioned that, although our focus was on cisplatin-indued nephrotoxicity, we found that the upward SMF did not have a combinational anti-cancer effect with cisplatin, while the downward SMF combined with cisplatin showed an improved antitumor effect in MDA-MB231 tumor-bearing mice, which was probably due to the improved overall physical state of the mice. In fact, the combinational effects of SMFs and cisplatin on cancer cells has been inconsistent in the literature. SMFs themselves have been shown to have some anti-cancer effects [37,38,39,40,41], and were also reported to be able to improve the anti-cancer efficacy of cisplatin [42,43,44,45]. However, the combination of cisplatin and an SMF does not always have a significant superimposed or synergistic effect [46,47]. For example, we previously found that a 1 T SMF does not increase the efficacy of cisplatin in HeLa, MCF7, HCT116, or CNE-2Z cancer cells [46]. Vergallo et al. studied the effect of 31.7–232 mT SMF in combination with cisplatin on human neuroblastoma SH-SY5Y, and found that two hours of treatment increased ROS and decreased cell viability, while 24 h of SMF treatment had the opposite effects [47]. The exact reasons for these effects are still unclear, and seem to be variable depending on the field parameters, cell type and time points. Moreover, all previous reported work on SMFs and cisplatin was carried out at the cellular level [42,43,44,45,46,47,48], but not at the animal level. 

## 5. Conclusions

In summary, our work reveals that moderate quasi-uniform SMFs at hundreds of mT can decrease cisplatin-induced nephrotoxicity in both in vitro and in vivo experiments. Cellular studies showed that SMF attenuated cisplatin damage to renal tubular cells by modulating oxidative stress. Animal studies revealed that both chronic and acute kidney injuries induced by cisplatin can be alleviated by SMFs, which was due to reduced oxidative stress, inflammation and cisplatin accumulation in the kidney. Therefore, our study suggests that moderate SMFs at hundreds of mT provided by permanent magnets could serve as low-cost physical tools to be developed as a possible adjuvant therapy to cisplatin chemotherapy to reduce cisplatin-induced kidney toxicity, which may also alleviate other side effects caused by drug-induced oxidative stress.

## Data Availability

All data generated and analyzed during the current study are available from the corresponding authors on reasonable request.

## Supplementary Materials

The following supporting information can be downloaded at: https://www.mdpi.com/article/10.3390/antiox12010073/s1, Figure S1: The effect of SMF treatment alone on HK-2 cell numbers; Figure S2: Tumor (A) and tumor weight (B) measured at the end of the experiment.; Figure S3: Representative HE (A) and PAS (B) staining of kidneys in high dose cisplatin-induced kidney injury experiments.; Figure S4: The effect of SMF treatment alone on Oct2 in HK-2 cells.
