# Supplementary material for: Static Magnetic Fields Protect against Cisplatin-Induced Kidney Toxicity
Source: Antioxidants (Basel). 2022 Dec 29;12(1):73. doi: 10.3390/antiox12010073 (PMC9854588; doi:10.3390/antiox12010073)
Supplement: Supplementary file 1 [file antioxidants-12-00073-s001.zip › antioxidants-2084499-supplementary.pdf]

Supplementary Materials

Article

Static Magnetic Fields Protect against Cisplatin-Induced Kidney Toxicity

Xin Yu [1,2], Xinmiao Ji [1], Yixiang Fan [1,2], Biao Yu [1], Xinyu Wang [1,3], Chuanlin Feng [1,2], Lei Zhang [1], Chao Song [1,*] and Xin Zhang [1,2,3,4,*]

1 High Magnetic Field Laboratory, Hefei Institutes of Physical Science, Chinese Academy of Sciences, Hefei 230031, China
2 Science Island Branch of Graduate School, University of Science and Technology of China, Hefei 230036, China
3 Institutes of Physical Science and Information Technology, Anhui University, Hefei 230601, China
4 International Magnetobiology Frontier Research Center, Science Island, Hefei 230036, China
* Correspondence: chaosong@hmfl.ac.cn (C.S.); xinzhang@hmfl.ac.cn (X.Z.)

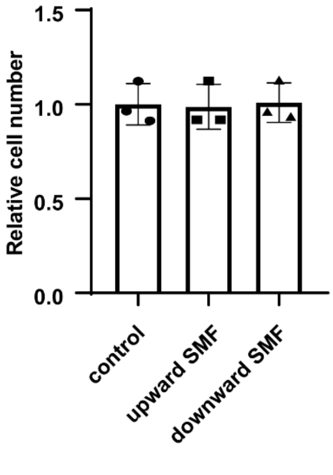

Figure S1: The effect of SMF treatment alone on HK-2 cell numbers. The HK-2 cells were cultured in cell culture dishes for 24 hours before they were exposed to upward or downward SMFs for 24 hours. They were then collected and counted by flow cytometry. The experiments were repeated for three times and no statistical significance were found between the three conditions.

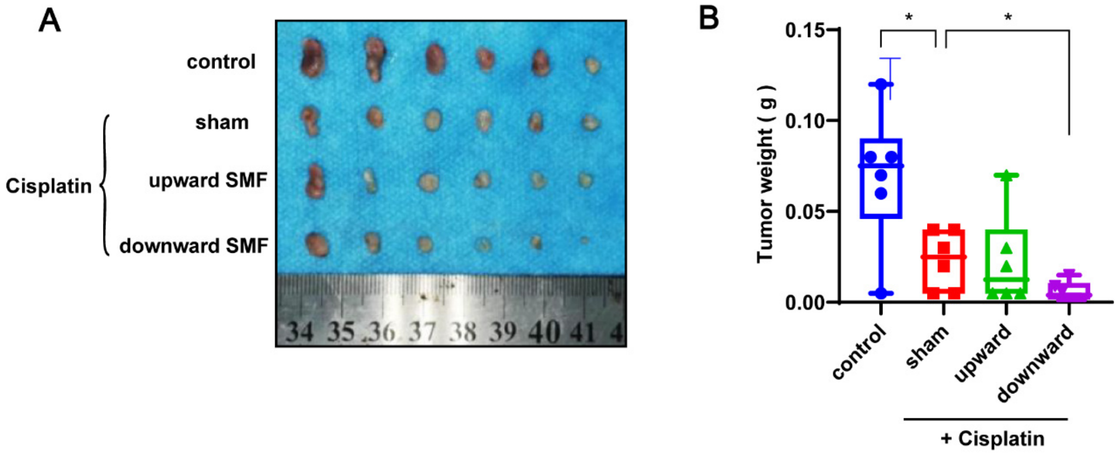

Figure S2: Tumor (A) and tumor weight (B) were measured at the end of the experiment. Data are represented as the mean ± SD. For those that have statistical significance, we label them as * $p < 0.05$.

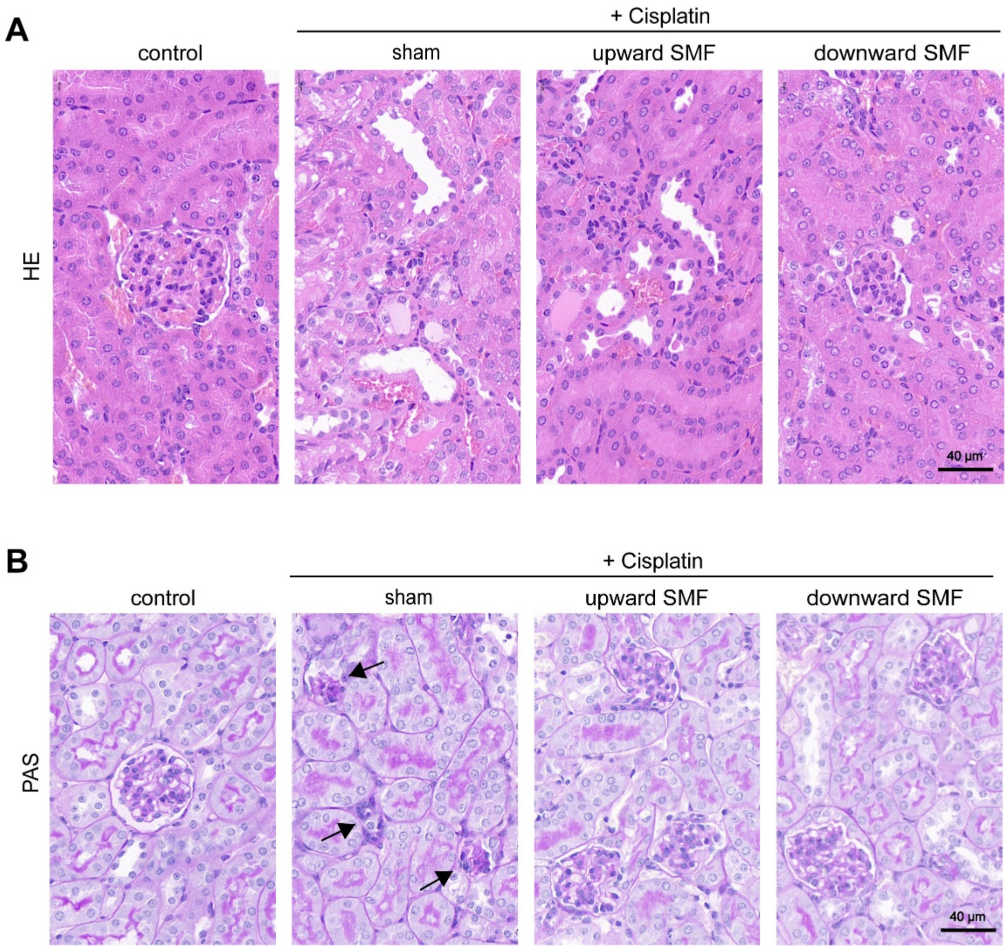

Figure S3: Representative HE (A) and PAS (B) staining of kidney in high dose cisplatin-induced kidney injury experiments. The arrows represent glomerular atrophy.

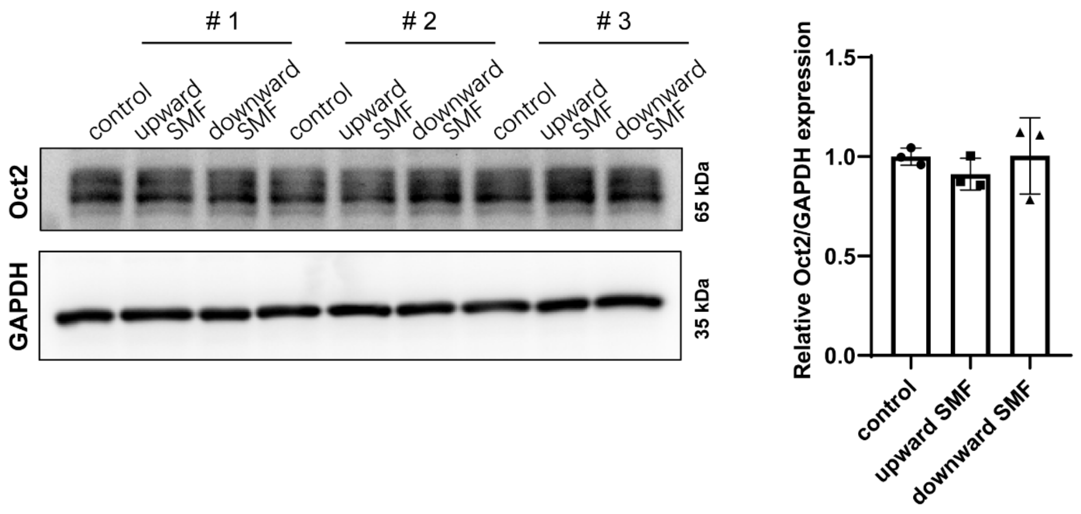

Figure S4: The effect of SMF treatment alone on Oct2 in HK-2 cells. The HK-2 cells were cultured in 35 mm culture dish for 24 hours, before they were treated with upward or downward SMFs for 24 hours and subjected to Western blot analysis. The experiments were repeated for three times and no statistical significance were found between the three conditions.
